# Supplementary material for: Antibiotic Treatment in Anopheles coluzzii Affects Carbon and Nitrogen Metabolism
Source: Pathogens. 2020 Aug 21;9(9):679. doi: 10.3390/pathogens9090679 (PMC7558193; doi:10.3390/pathogens9090679)
Supplement: Supplementary file 1 [file pathogens-09-00679-s001.pdf]

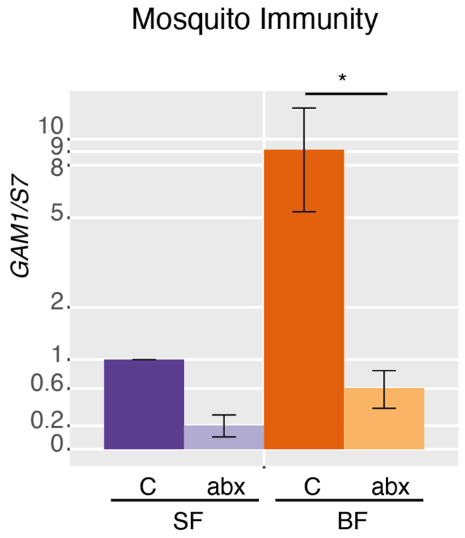

Figure S1. RT-qPCR analysis of GAM1 immune gene expression. Quantification of GAM1 in the gut of sugar-fed (SF) and blood-fed (BF) mosquito carrying a conventional (C) microbiota or treated with antibiotics (abx). Data show the average ratios of two independent replicates, and error bars represent the standard error. Tukey test were used to determine the confidence interval, ns non significative, * $p < 0.05$, ** $p < 0.01$.

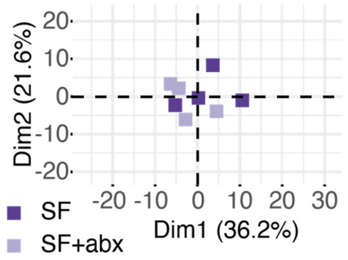

Figure S2. Principal Component Analysis (PCA) plot of the midgut metabolome. Only sugar-fed samples are included in the analysis.

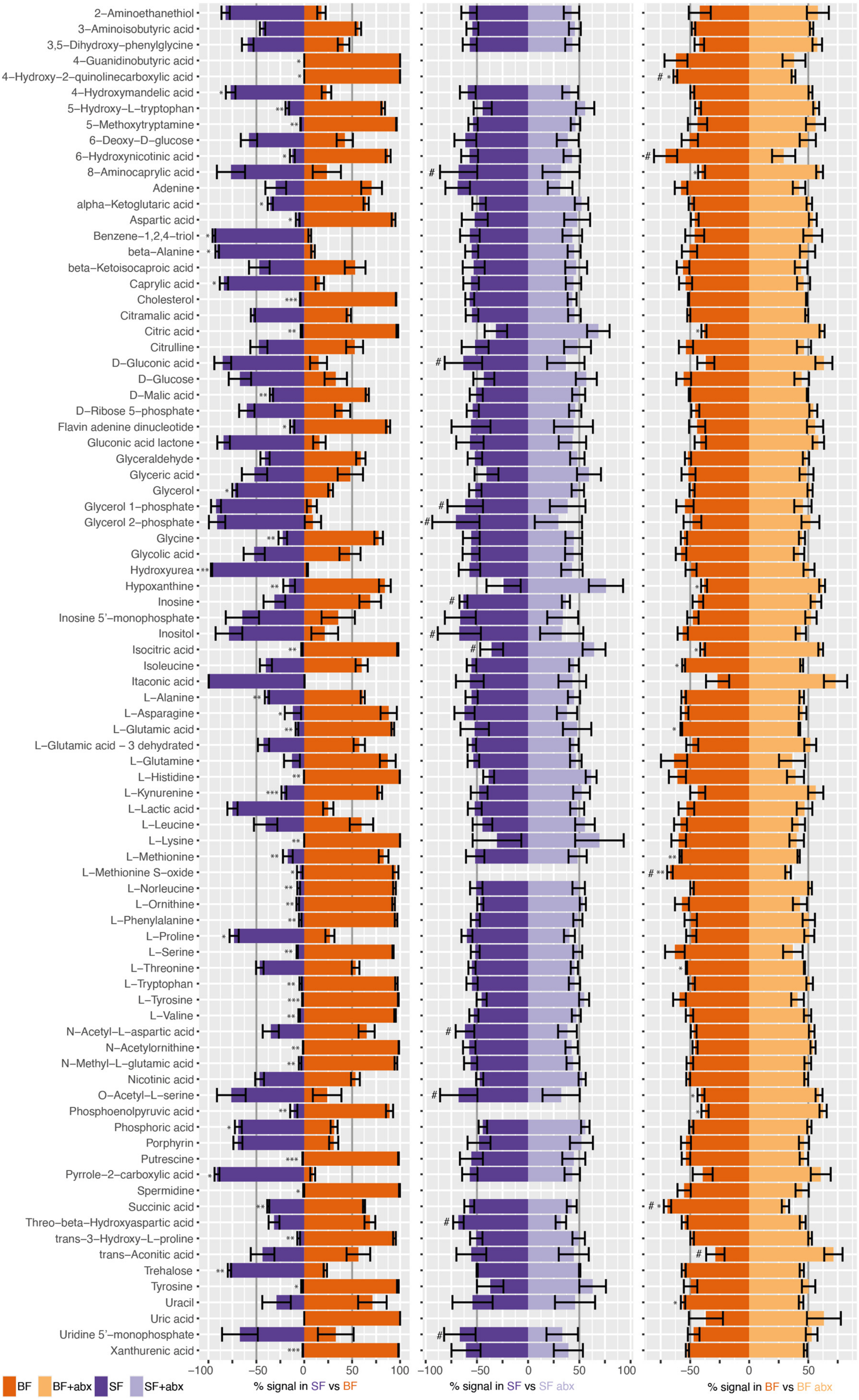

2−Aminoethanethiol
3−Aminoisobutyric acid
3,5−Dihydroxy−phenylglycine
4−Guanidinobutyric acid
4−Hydroxy−2−quinolinecarboxylic acid
4−Hydroxymandelic acid
5−Hydroxy−L−tryptophan
5−Methoxytryptamine
6−Deoxy−D−glucose
6−Hydroxynicotinic acid
8−Aminocaprylic acid
Adenine
alpha−Ketoglutaric acid
Aspartic acid
Benzene−1,2,4−triol
beta−Alanine
beta−Ketoisocaproic acid
Caprylic acid
Cholesterol
Citramalic acid
Citric acid
Citrulline
D−Gluconic acid
D−Glucose
D−Malic acid
D−Ribose 5−phosphate
Flavin adenine dinucleotide
Gluconic acid lactone
Glyceraldehyde
Glyceric acid
Glycerol
Glycerol 1−phosphate
Glycerol 2−phosphate
Glycine
Glycolic acid
Hydroxyurea
Hypoxanthine
Inosine
Inosine 5'−monophosphate
Inositol
Isocitric acid
Isoleucine
Itaconic acid
L−Alanine
L−Asparagine
L−Glutamic acid
L−Glutamic acid − 3 dehydrated
L−Glutamine
L−Histidine
L−Kynurenine
L−Lactic acid
L−Leucine
L−Lysine
L−Methionine
L−Methionine S−oxide
L−Norleucine
L−Ornithine
L−Phenylalanine
L−Proline
L−Serine
L−Threonine
L−Tryptophan
L−Tyrosine
L−Valine
N−Acetyl−L−aspartic acid
N−Acetylornithine
N−Methyl−L−glutamic acid
Nicotinic acid
O−Acetyl−L−serine
Phosphoenolpyruvic acid
Phosphoric acid
Porphyrin
Putrescine
Pyrrole−2−carboxylic acid
Spermidine
Succinic acid
Threo−beta−Hydroxyaspartic acid
trans−3−Hydroxy−L−proline
trans−Aconitic acid
Trehalose
Tyrosine
Uracil
Uric acid
Uridine 5'−monophosphate
Xanthurenic acid
−100 −50 0 50 100
% signal in SF vs BF
−100 −50 0 50 100
% signal in SF vs SF abx
−50 0 50
% signal in BF vs BF abx
BF BF+abx SF SF+abx

Figure S3. Bar charts representing the comparative percentage of signal from different conditions. Data represent the average percentage from 4 independent replicates and error bars show the standard error. t-test were used to determine statistical significance, * −p < 0.05, ** −p < 0.01, *** −p < 0.001. # indicate metabolites which are consistently regulated at least 1.7-fold (ratio >1.7 or <0.588) in at least 3 of the 4 replicates by the antibiotic treatment.

Table S1. Primers sequences for qPCR.

Primer name	Forward/ Reverse	Gene Name	ACOM #	Sequence
Cec1 F qPCR	F	*Cecropin1*	042420	TCATCTTTGTCGTGCTGGCA
CEC1 R qPCR	R	*Cecropin1*	042420	TCTTCAGCCGTCCCGCT
Gam1_F	F	*Gambicin 1*	024216	CTATCTCAACCGGAAGGGCG
Gam1_R	R	*Gambicin 1*	024216	GCCAAACTTTCGCTTGCAGT
PGRPLB_qPCR F	F	*PGRPLB*	033796	GAGTATGGTGTCCGGAATGG
PGRPLB_qPCR R	R	*PGRPLB*	033796	GGCCAGGTTTTGATCTCCTC
S7-F	F	*S7*	041783	GTGCGCGAGTTGGAGAAGA
S7-R	R	*S7*	041783	ATCGGTTTGGGCAGAA TGC
16s F qPCR	F	Generic bacterial 16S	N/A	TCCTACGGGAGGCAGCAGT
16s R qPCR	R	Generic bacterial 16S	N/A	GGACTACCAGGGTATCTAATCCTGTT
